# Modulation of HIV-1 capsid multimerization by sennoside A and sennoside B via interaction with the NTD/CTD interface in capsid hexamer

**DOI:** 10.3389/fmicb.2023.1270258

**Published:** 2023-09-25

**Authors:** Da-Wei Zhang, Xiao-Shuang Xu, Rui Zhou, Zhiguo Fu

**Affiliations:** ^1^Institute of Bioinformatics and Medical Engineering, School of Electrical and Information Engineering, Jiangsu University of Technology, Changzhou, China; ^2^Cancer Center, Union Hospital, Tongji Medical College, Huazhong University of Science and Technology, Wuhan, China; ^3^Institute of Radiation Oncology, Union Hospital, Tongji Medical College, Huazhong University of Science and Technology, Wuhan, China; ^4^Department of Orthopedics, Changzhou Hospital of Traditional Chinese Medicine, Changzhou, China

**Keywords:** HIV-1 capsid, capsid assembly, hexamer, drug screening, biolayer interferometry

## Abstract

Small molecules that bind to the pocket targeted by a peptide, termed capsid assembly inhibitor (CAI), have shown antiviral effects with unique mechanisms of action. We report the discovery of two natural compounds, sennoside A (SA) and sennoside B (SB), derived from medicinal plants that bind to this pocket in the C-terminal domain of capsid (CA CTD). Both SA and SB were identified via a drug-screening campaign that utilized a time-resolved fluorescence resonance energy transfer assay. They inhibited the HIV-1 CA CTD/CAI interaction at sub-micromolar concentrations of 0.18 μM and 0.08 μM, respectively. Mutation of key residues (including Tyr 169, Leu 211, Asn 183, and Glu 187) in the CA CTD decreased their binding affinity to the CA monomer, from 1.35-fold to 4.17-fold. Furthermore, both compounds induced CA assembly *in vitro* and bound directly to the CA hexamer, suggesting that they interact with CA beyond the CA CTD. Molecular docking showed that both compounds were bound to the N-terminal domain (NTD)/CTD interface between adjacent protomers within the CA hexamer. SA established a hydrogen-bonding network with residues N57, V59, Q63, K70, and N74 of CA1-NTD and Q179 of CA2-CTD. SB formed hydrogen bonds with the N53, N70, and N74 residues of CA1-NTD, and the A177and Q179 residues of CA2-CTD. Both compounds, acting as glue, can bring αH4 in the NTD and αH9 in the CTD of the NTD/CTD interface close to each other. Collectively, our research indicates that SA and SB, which enhance CA assembly, could serve as novel chemical tools to identify agents that modulate HIV-1 CA assembly. These natural compounds may potentially lead to the development of new antiviral therapies with unique mechanisms of action.

## Introduction

Approximately 38 million individuals worldwide currently live with HIV-1, with 40.1 million deaths due to AIDS-related illnesses since 1981 (UNAIDS, 2021). Infection with HIV-1 is incurable, except in three cases of HIV-1 cure (Hütter et al., 2009; Allers et al., 2011; Gupta et al., 2019, 2020). In the past three decades, the use of combination antiretroviral therapy (cART) has led to a substantial reduction in mortality among those living with HIV-1 infection, which has effectively transformed AIDS from a fatal pandemic to a chronic and manageable disease (Trickey et al., 2017). Although cART has been highly successful, its efficacy can be severely compromised by partial adherence to treatment, emergence of drug resistance, and establishment of HIV-1 latency (Chun et al., 1997; Finzi et al., 1997; Wong et al., 1997; Siliciano et al., 2003; Zhang, 2018). Therefore, the identification of new drugs that target alternative targets is of high priority.

HIV-1 particle assembly involves a two-step process. The initial assembly stage forms a spherical shell of immature virions made up of Gag and Gag-Pol polyproteins in uncleaved form, and the second stage results in a mature conical core built by capsid proteins (CA) released by proteolytic processing of Gag (Ganser et al., 1999; Freed, 2015). The assembly of the immature Gag shell and the mature capsid relies on CA, which is involved in multiple stages of HIV-1 replication. Therefore, CA represents an attractive target for therapeutic intervention (Thenin-Houssie and Valente, 2016; Carnes et al., 2018). However, CA performs its functions through protein–protein interactions, which have historically been considered a significant challenge in small-molecule drug interventions (Scott et al., 2016). Fortunately, in the past 15 years, various efforts have been made to identify compounds that can bind to HIV-1 CA, resulting in the discovery of a few such molecules, such as PF-3450074 (PF74), GS-6207, MKN-1A, I-XW-053, BD-1, CAI, BM-1, HCB, and ACAi-028 (Sticht et al., 2005; Blair et al., 2010; Kortagere et al., 2012; Lemke et al., 2012; Jacques et al., 2016; Link et al., 2020; Chia et al., 2021; Kobayakawa et al., 2021). Among these, lenacapavir (GS-6207) has been approved by the FDA for medical use (Mushtaq and Kazi, 2023), which strongly indicates that HIV-1 CA is an effective target for the development of new antiviral medications.

**Figure 1:**
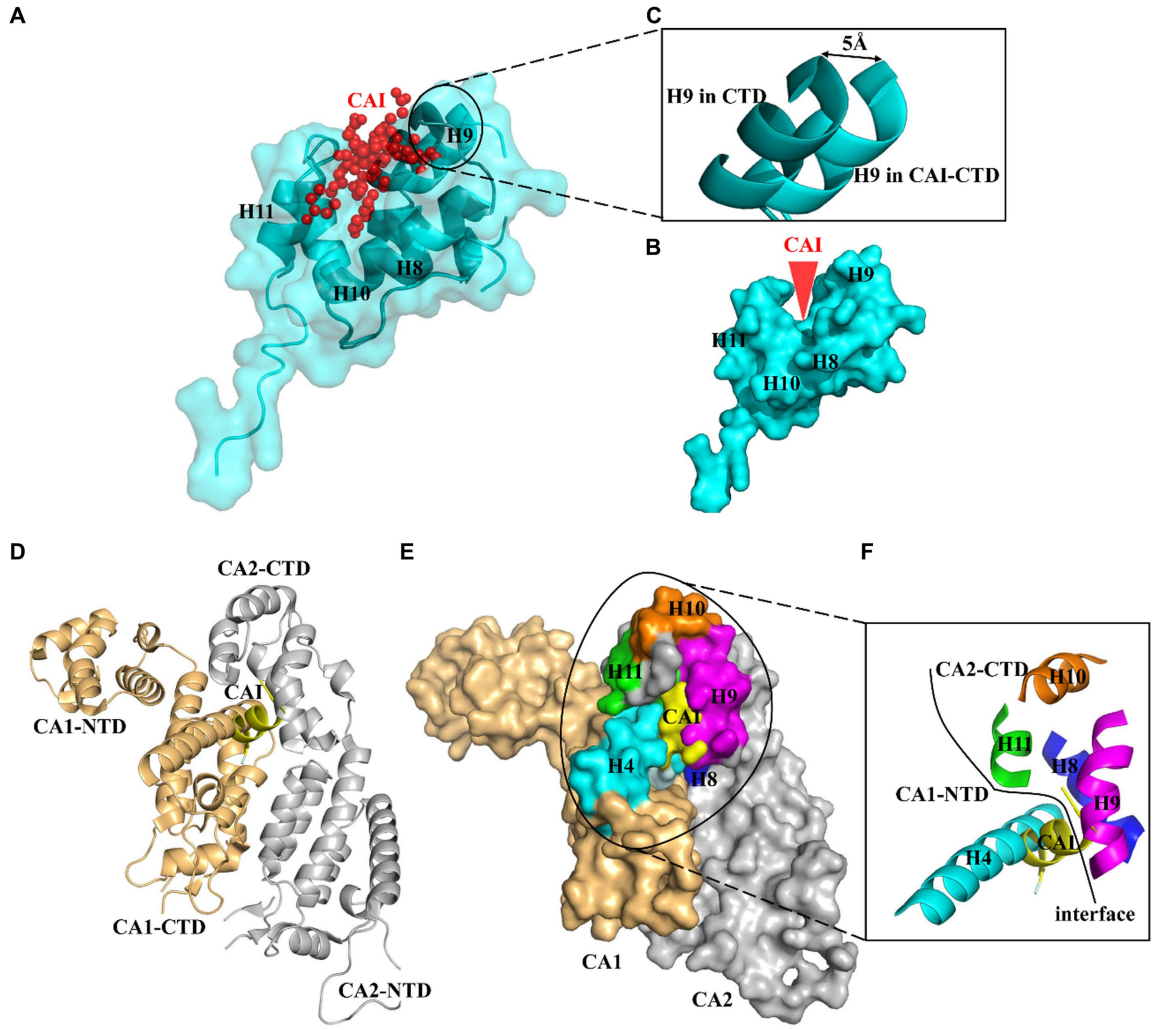
Profile of CAI-binding pocket. **(A)** The peptide CAI inserts as an amphipathic α-helix into a conserved hydrophobic groove formed by three helices (H8, H9 and H11) of CA CTD and wedges this cavity open. **(B)** CAI wedges its binding pocket open and **(C)** induces a slight opening of the cavity with the displacement being about 6 A° at the N terminus of helix 9 (H9). **(D)** CAI also blocks the CA NTD/CA CTD interaction between neighboring CA molecules. This interaction surfaces are mapped to helix 4 in the CA NTD and helices 8 and 9 in the CA CTD. **(E)** and **(F)** CAI binds to the same interaction surfaces occupied by helix 4.

The CA protein comprises two independently folded domains, an N-terminal domain (NTD) and a C-terminal domain (CTD), which are connected by a flexible linker (Momany et al., 1996). A dodecapeptide, CAI, which was identified through phage display screening, has been found to be capable of inhibiting the *in vitro* assembly of immature-like as well as mature-like capsid particles (Sticht et al., 2005). Structural analysis revealed that the peptide was bound to a conserved cavity located in the CA CTD. This groove plays a crucial role in regulating the protein conformation required for the formation of infectious virions (Ternois et al., 2005). Recent research has suggested two reasons for why CAI disrupts CA assembly (Ternois et al., 2005; Bartonova et al., 2008). First, the amphipathic alpha-helix structure of the CAI peptide allows it to be inserted into a conserved hydrophobic pocket composed of three helices (H8, H9, and H11) of the CA CTD, forcing the cavity to remain open. This structural change causes loss of the necessary flexibility in the CA protein and restricts it to a rigid conformation that cannot support particle formation (Figures 1A–C). Second, CAI directly impeded the CA1-NTD/CA2-CTD interaction, which is critical for the assembly of the mature virion (Figures 1D–F). Therefore, this groove on the CA CTD potentially serves as a target for screening small molecules that allosterically lock the CA CTD in an inactive conformation, thereby disrupting CA assembly (Sticht et al., 2005; Ternois et al., 2005). Since then, various HIV-1 inhibitors targeting this conserved pocket have been identified, including small molecules and stapled peptides (Zhang et al., 2008, 2011; Zhang et al., 2013; Machara et al., 2016). The potency of these inhibitors suggests that the CAI-binding pocket is a promising target for the development of antiviral therapies against HIV-1.

This study aimed to screen an FDA-approved drug library and identify novel compounds that could bind to the CAI-binding cavity. Sennoside A (SA) and sennoside B (SB) are potential modulators of CA assembly. They accelerated the CA assembly process by targeting the preassembled CA hexamers. It is speculated that both compounds bind to the interface between CA subunits, facilitating CA assembly. As a result, they may serve as promising candidates for developing HIV-1 CA assembly modulators.

## Materials and methods

### Chemicals and reagents

C-terminally biotinylated CAI peptide (ITFEDLLDYYPGGGSK-biotin) and unmodified CAI were synthesized by GL Biochem (Shanghai) Ltd. The Discovery Probe FDA-approved Drug Library was purchased from the National Compound Resource Center (Shanghai, China), whereas HTRF detection agents and white 384-well plates were purchased from PerkinElmer (Boston, MA, United States). Black 96-well plates were sourced from Greiner Bio-One (Darmstadt, Germany) and streptavidin-coated biosensors were purchased from Sartorius AG (Goettingen, Germany). Affinity chromatography resin for the purification of His-tagged and GST-tagged proteins was procured from Changzhou Smart-Lifesciences Biotechnology Co. Ltd. (Changzhou, China), while other reagents were obtained from Amresco (Solon, USA). Sennoside A (purity ≥99%) and sennoside B (purity ≥99) were commercially available from the supplier MedChem Express (Shanghai, China).

### Protein expression and purification

DNA encoding the CA CTD was cloned into BamHI-NotI sites of prokaryotic expression vector pGEX-4 T-1 with a N-terminal glutathione S-transferase (GST) tag. Recombinant CA CTD protein was expressed in *E. coli* BL21(DE3) and purified as previously described (Thenin-Houssier et al., 2016).

DNA encoding the wild type (WT) CA was cloned into Nde I-BamHI sites of prokaryotic expression vector pET11a. Expression and purification of WT CA proteins were performed using the same procedure as previously described (Lanman et al., 2002).

The plasmid expressing a quadruple mutant (A14C/E45C/W184A/M185A) CA (4Mu CA) was constructed by introducing the quadruple mutations to CA gene in plasmid for expression of prokaryotic WT *CA.* Expression of WT CA proteins were followed the same scheme as WT *CA.* The purification protocol for 4Mu CA followed the same scheme as WT CA, except that 200 mM β-mercaptoethanol was included in all buffers and ammonium sulfate fractionation was performed at 30% saturation.

After purification, CA proteins were dialyzed overnight against 20 mM Tris (pH 8.0) containing 5 mM β-ME. Subsequently, the purified proteins were concentrated to approximately 30 mg/mL, divided into aliquots, flash frozen in liquid nitrogen, and stored at −70°C until further analysis.

### Primary drug screening

The Discovery Probe FDA-approved Drug Library comprised 1,971 compounds, which were available as stock solutions at 10 mM concentrations in DMSO. Primary screening of these compounds was conducted using a previously established HTRF assay (Zhang et al., 2019). Briefly, compound (1 μL), CAI-biotin peptide (2 μL) and GST-CA CTD (2 μL) were dispensed into 384-well microplates. After 30 min incubation at room temperature, 5 μL premixed HTRF agents were added. After an additional 1 h incubation at room temperature, the HTRF signal was acquired by reading the plates in a PerkinElmer Envision Multilabel Plate Reader. Data were analyzed and visualized using GraphPad Prism (version 5.0). To ensure the reproducibility of the results, duplicate assays were conducted for all drugs. Quality control measures were implemented, and only plates with a Z’-factor greater than 0.5 were included in the subsequent analysis. The quality of the assay was determined by calculating the Z’-factor using the following equation: Z’ = 1 - (3 × SD_max_ + 3 × SD_min_) / |μ_max_ - μ_min_|, where SD_max_ and SD_min_ represent the standard deviations of the positive and negative control measurements, respectively, and μ_max_ and μ_min_ represent the means of the respective positive and negative signal controls (Zhang et al., 1999).

### Assembly of recombinant CA proteins

To monitor the *in vitro* assembly of HIV-1 CA in the presence and absence of hit compounds, changes in sample absorbance over time at 350 nm were measured, as previously described (Lanman et al., 2002; Tang et al., 2003; Xu et al., 2018). To initiate the assay, 1 μL of 5 mM SA or SB was added to 74 μL of buffer composed of a mixture of 5 M NaCl and 200 mM NaH_2_PO_4_ (pH 8.0) in a ratio of 2:1 by volume. Subsequently, 25 μL of CA (120 μM) was added to each well and DMSO served as a vehicle control. After a 2-min equilibration period, sample absorbance values were measured at 350 nm every 60 s for 39 min using a Perkin Elmer Envision microplate reader and were corrected by subtracting the absorbance values of a blank sample without sodium chloride. The resulting data were analyzed using GraphPad Prism 5.0.

### Biotinylation of HIV-1 CA

To obtain HIV-1 hexameric CA, the recombinant 4Mu CA was used, which have been shown to promote stable hexameric unit formation in a previous study (Pornillos et al., 2009, 2010). Briefly, Crosslinked CA hexamers were prepared by sequential dialysis of 30 mg/mL 4Mu CA into assembly buffer (NaH_2_PO_4_-Na_2_H_2_PO_4_, pH8.0, 2.5 M NaCl) containing 200 mM β-ME, assembly buffer with 0.2 mM β-ME, and, finally, 20 mM Tris (pH 8.0). Each dialysis step was performed at 4°C, for at least 8 h. Biotinylation of both wild-type (WT) and mutant HIV-1 CA monomers as well as the 4Mu CA hexamer, was achieved using EZ-Link Sulfo-NHS-biotin reagents, according to the manufacturer’s instructions (Thermo Fisher).

### Biolayer interferometry assay

To confirm the affinity of the compound for HIV-1 CA, biolayer interferometry (BLI) experiments were conducted using an Octet96 system at 25°C. Streptavidin biosensor (SA) sensors were immersed in phosphate-buffered saline (pH 7.5) with Tween 20 (0.02%) and DMSO (1%) for at least 10 min prior to the run. Biotinylated proteins were loaded onto the tips of SA sensors. The double reference subtracted method was conducted, as previously described, to determine three affinity constants: k_on_, k_off_, and K_D_ (Zhang et al., 2023).

### Molecular docking

Four crystal structures, including CAI-bound CA CTD (PDB ID:2BUO), native CA hexamer (4XFX), PF74 bound native CA hexamer (4XFZ), and GS-6207 bound CA_A14C/E45C/W184A/M185A_ hexamer (6VKV) were used for molecular docking. All molecular simulations were performed using the SwissDock on-line server (Grosdidier et al., 2011a,b), and the results were visualized using PyMOL.^1^

### Anti-HIV-1 activity assay

Anti-HIV-1 activity was determined by an assay based on virus-induced cytopathic effects (CPE) (Zhang et al., 2020). Briefly, C8166 cells were infected with HIV-1 at a multiplicity of infection (M.O.I) of 0.03, with DMSO or diluted SA or SB in 96-well plates. After 72 h, the CPE was determined by light microscopy and the 50% effective concentrations (EC_50_) were calculated. The cytotoxicity of the compounds was determined by a tetrazolium dye (MTT) based assay. C8166 cells were co-incubated with diluted SA or SB in 96-well plates. After 3 days, cell viability was determined using MTT, and the 50% cytotoxicity concentration (CC_50_) was calculated. The selectivity index (SI) was calculated using the formula: SI = CC_50_/EC_50_, which indicates the magnitude between cytotoxic and effective concentrations of the compound. Tenofovir disoproxil fumarate (TDF) was used as a positive control.

### Statistical analysis

Data are presented as means ± SEM. Paired-test was used for statistical significance determination using the GraphPad Prism 5.0. Significance was set at *p* < 0.05.

### Declarations

Our study does not include any human research participants and animals.

## Results

### Screening of hit compounds targeting CA CTD/CAI interaction

**Figure 2:**
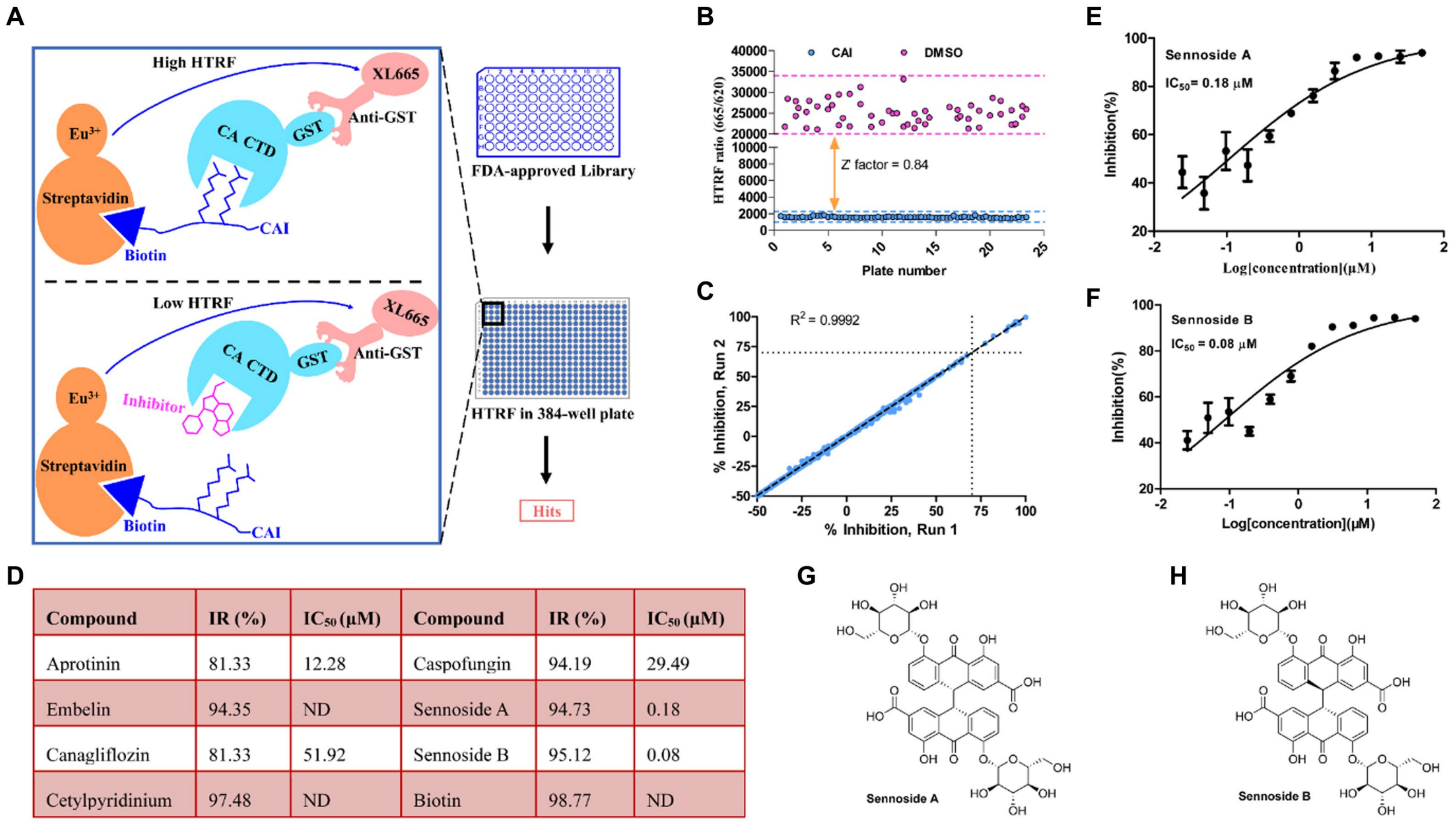
Screening for inhibitors of CA CTD-CAI interaction. **(A)** Illustration of the HTRF-based high-throughput screening platform for CA CTD-CAI interaction inhibitors. **(B)** Screening validation. The Z-factor of 0.84 demonstrates excellent assay. **(C)** Correlation plot of the compound activities for the duplicate runs. **(D)** Hits obtained from the primary screening of the FDA-approved Library for the disruption of the CA CTD–CAI interaction. Dose–response curves of Sennoside A **(E)** and Sennoside B **(F)**. Panels **(E,F)** results are averages ± standard deviation for *n* = 3 independent experiments. Chemical structures of Sennoside A **(G)** and Sennoside B **(H)**. ND is the abbreviation of no data, and IR% is the inhibitory rate (100%).

HTRF is a highly effective platform for conducting high-throughput screening (HTS) (Rectenwald et al., 2019). We employed this method to create an assay to identify small-molecule drugs that could disrupt the interaction between peptide CAI and CA CTD (Zhang et al., 2019). The screening approach and workflow are shown in Figure 2A. We screened a library of 1,971 small molecules, all of which were FDA-approved. Each compound was tested in duplicate at a concentration of 50 μM. To evaluate the quality of the screening process, we used the Z′-factor based on the signals between the 1.0% DMSO (negative control) and 50 μM CAI-treated groups. The average Z-factor for all screenings was 0.84, indicating that the results were reliable (Figure 2B). Screening of 1,971 compounds also demonstrated high reproducibility, with a correlation coefficient (R) of 0.9992 (Figure 2C). Eight hits with over 70% inhibition (Figure 2D) were identified including biotin, an assay interference compound, which was excluded from further investigation. The remaining compounds were further validated through dose–response analysis using the HTRF assay.

### Dose–response assessment

**Figure 3:**
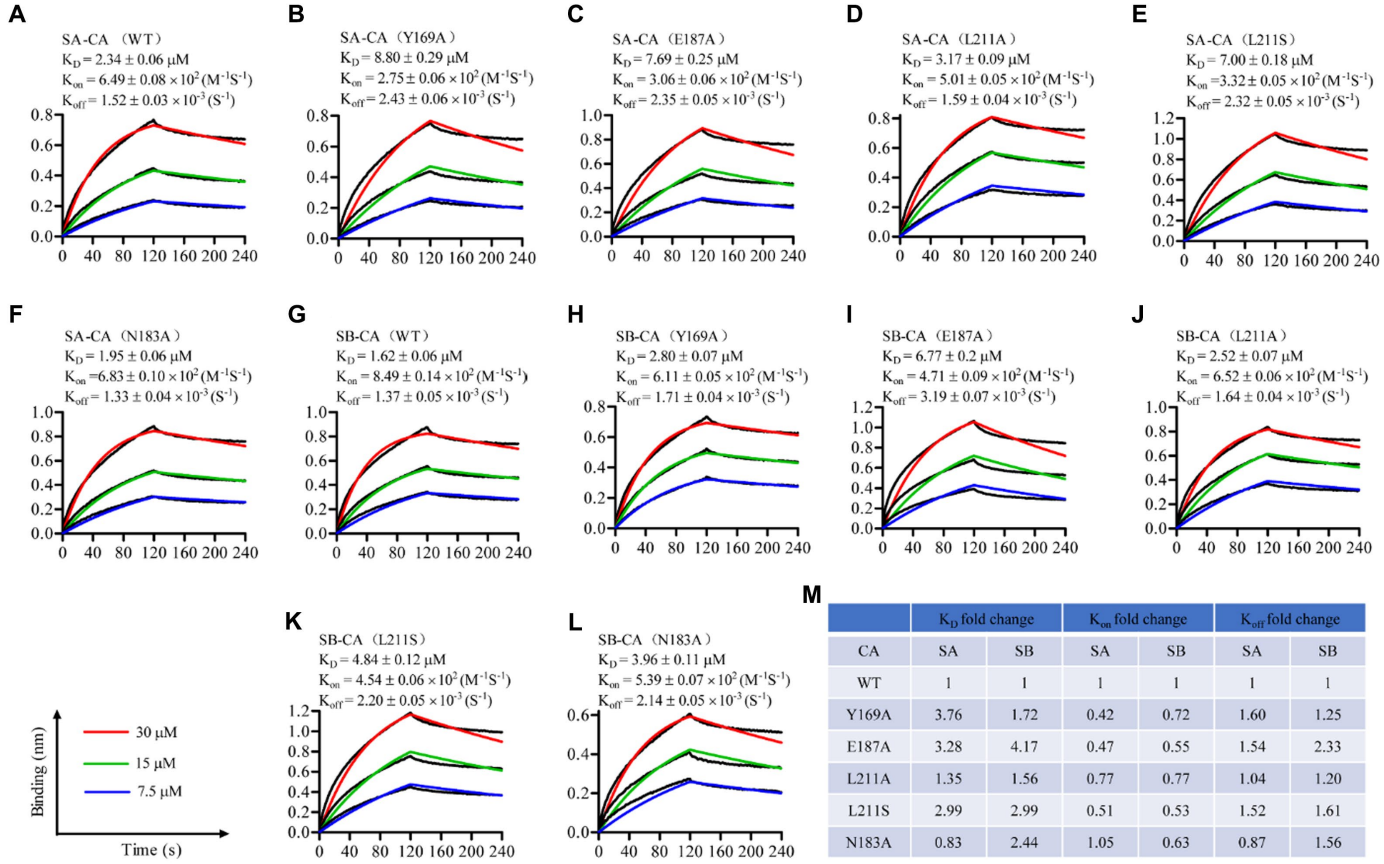
Sennoside A **(A–F)** and Sennoside B **(G–L)** binding to HIV-1 CA monomer. Representative binding sensorgrams illustrating interactions between Sennoside A or Sennoside B with wild-type (WT) and mutant HIV-1 CA monomers evaluated by bio-layer interferometry (BLI) detection. The biotin-conjugated protein was captured by streptavidin that was immobilized on a chip and tested for binding with gradient concentrations (7.5–30 μM) of SA or SB. Binding kinetics were evaluated using a 1:1 Langmuir binding model by ForteBio Data Analysis 9.0 software. The mean ± standard deviation (SD) equilibrium dissociation constant (K_D_), association rate constant (K_on_), and dissociation rate constant (K_off_) values were determined from three independent experiments with comparable results. **(M)** Fold change of the K_D_, K_on_, and K_off_ values for the mutant CA monomers compared to WT *CA.*

Figure 3A and 1.62 μM (Figure 3G), respectively. These results indicate that both compounds bind directly to CA and compete with CAI for binding to the CA CTD. Therefore, based on these findings, SA and SB were chosen as the preferred candidates for subsequent analyses in further experiments.

Seven hit compounds were repurchased and subjected to a 12-point two-fold serial dilution, from 50 to 0.024 μM. Of these seven compounds, five were found to be effective in dose–response assays (Figure 2D). Specifically, two of the compounds, namely sennoside A (SA) and sennoside B (SB), exhibited strong inhibitory activity with IC_50_ values of 0.18 μM (95% confidence interval [CI], 0.15 μM–0.23 μM) (Figure 2E) and 0.08 μM (95% confidence interval [CI], 0.06 μM–0.12 μM) (Figure 2F), respectively. Their chemical structures are shown in Figures 2G,H. To further confirm their affinity for CA, both SA and SB were analyzed using the BLI assay. This assay was conducted using three different concentrations of the compounds to determine their binding to monomeric *CA.* The results showed that both compounds interacted directly with the CA monomer with a K_D_ value of 2.34 μM (Figure 3A) and 1.62 μM (Figure 3G), respectively. These results indicate that both compounds bind directly to CA and compete with CAI for binding to the CA CTD. Therefore, based on these findings, SA and SB were chosen as the preferred candidates for subsequent analyses in further experiments.

### Mutations of residues in the CAI-binding pocket decrease affinity between SA or SB and HIV-1 CA monomer

To identify the binding site of SA and SB on the CA protein, several key residues within the CA protein, including Y169, N183, E187, and L211, which are critical for the binding of CAI to the CA CTD, were selected for mutagenesis experiments. We introduced the mutations Y169A, N183A, E187A, and L211A/S into each plasmid and purified the mutant proteins from *E. coli* (Bartonova et al., 2008). Subsequently, we used BLI to determine the equilibrium dissociation (K_D_), association rate (K_on_), and dissociation rate (K_off_) constants for the binding of SA and SB to the mutant CA monomers. For SA, the Y169A, E187A, and L211S substitutions affected both the K_on_ and K_off_ values of SA, resulting in an approximately 3- to 3.7-fold decrease in the binding affinity of the two natural products to the CA monomer (Figures 3A–C,M). The L211A substitution resulted in a 1.35-fold reduction in the K_D_ value, primarily because of a similar reduction in K_off_, whereas the K_on_ values of SA association for L211A and WT CA showed a small difference (Figures 3A,D,M). In contrast, the N183A mutation led to a slight decrease in the K_D_ value with reduced levels of K_off_, although the K_on_ values of SA association for N183A and WT CA were similar. In the case of SB, all five substitutions affected both the K_on_ and K_off_ constants of the compound, leading to a 1.72- to 4.17-fold decrease in the binding affinity to the CA monomer (Figures 3G–M). The E187A substitution had the most pronounced effect on the SB equilibrium dissociation (K_D_), compared to other substitutions, such as Y169A, L211A, L211S, and N183A substitutions had the most pronounced effect on SB equilibrium dissociation (K_D_). Taken together, these results provide compelling evidence that SA and SB bind directly to the CA protein as direct competitors of the CAI-CA CTD interaction.

### SA and SB increase CA multimerization *in vitro*

**Figure 4:**
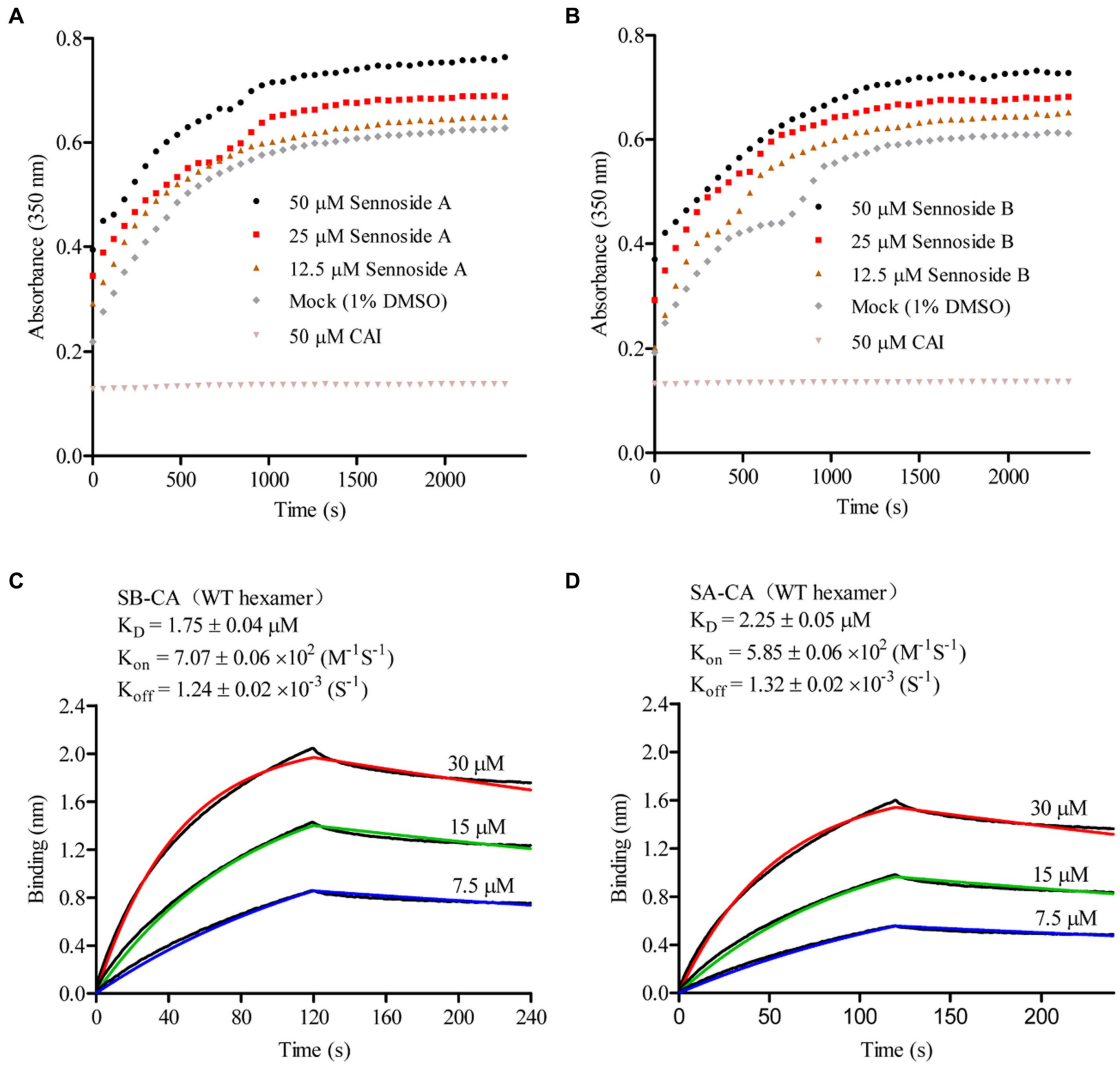
Sennoside A and Sennoside B induced CA multimerization *in vitro*. **(A–B)** Light scattering (absorbance at 350 nm) responses showing the rate and extent of *in vitro* CA (20 μM) assembly in 2.5 M NaCl, in absence of compounds (mock) and the presence of Sennoside A and Sennoside B or CAI. Data are representative of three independent experiments. **(C–D)** Representative binding sensorgrams depicting the interaction of SA or SB with CA hexamer assessed by bio-layer interferometry (BLI) detection.

Under high ionic strength conditions, recombinant CA can spontaneously oligomerize and form open-ended helical tubes consisting of repeating CA hexamers. To investigate the impact of SA and SB on CA tube assembly, we monitored *in vitro* CA assembly in the presence and absence of SA and SB by measuring changes in light absorbance over time in 2.5 M NaCl. CA (25 μM) for *CA.* In contrast to the CAI peptide (positive control), which inhibited CA assembly *in vitro*, both SA and SB increased *in vitro* CA assembly in a dose-dependent manner (Figures 4A,B). We used BLI to determine the K_D_ values of SA and SB in the HIV-1 CA hexamer. Figures 4C,D presented the results, which indicated that they bound firmly to the hexameric CA, with K_D_ values of 1.75 μM and 2.25 μM, respectively. These findings suggest that SA or SB increases capsid *in vitro* assembly by binding to the CA hexamer.

### SA and SB bind to NTD-CTD intersubunit interface within the CA hexamer

**Figure 5:**
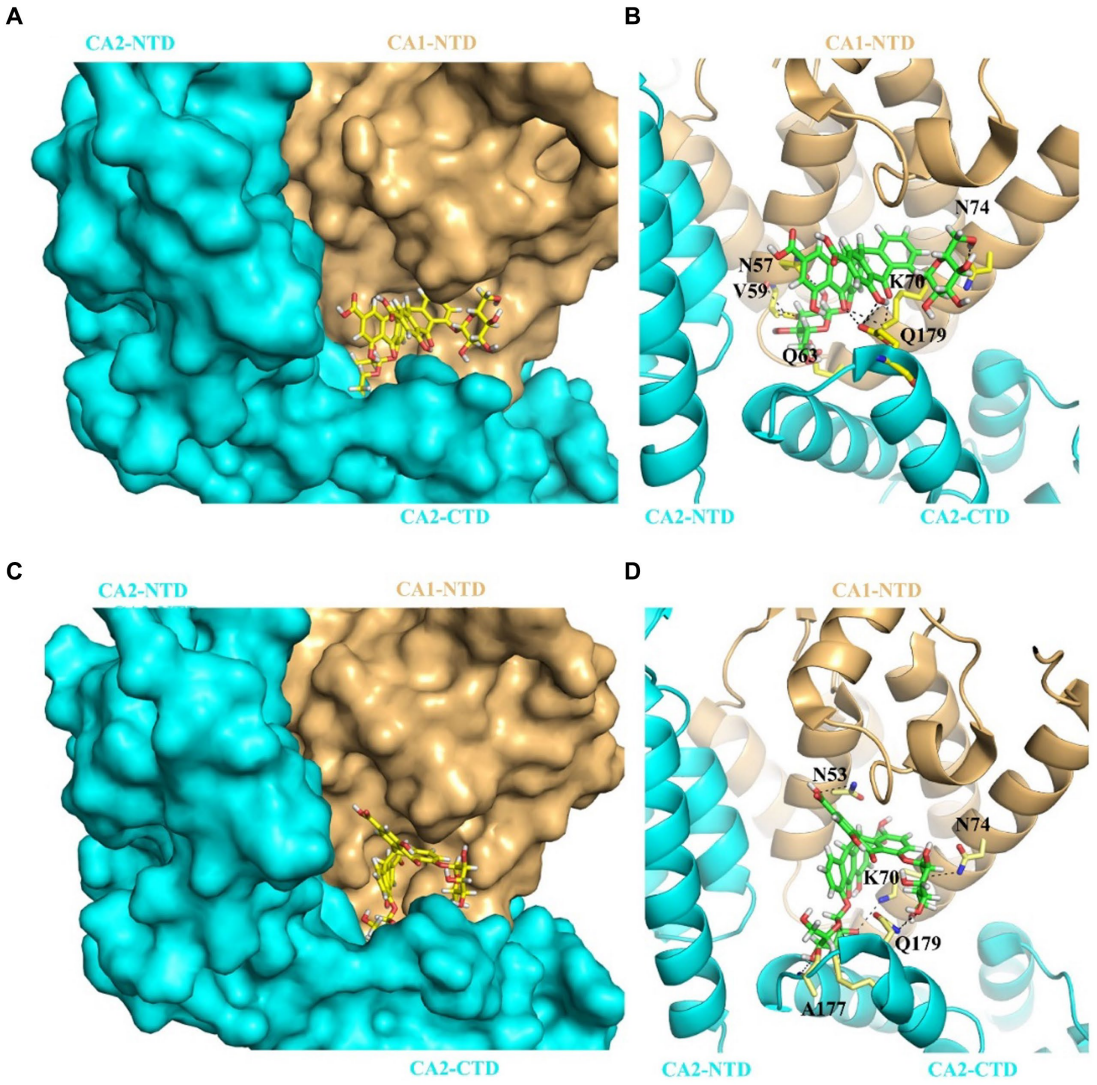
Predicted binding mode for SA or SB interaction with of CA hexamer. **(A)** The complex structure of SA bound to the CA_A14C/E45C/W184A/M185A_ hexamer (PDB: 6VKV). SA binds at the pocket formed by two adjacent CA protomer CA1 (pale yellow) and CA2 (cyan). Relative positions of each CA subunits are indicated as CA1-NTD, CA2-NTD, and CA2-CTD, respectively. **(B)** Cartoon representation of the structure indicating SA’s interactions with the two subunits that form the binding pocket, CA1 and CA2. Hydrogen bonds are denoted by black dashed lines. **(C)** The complex structure of SB bound to the CA_A14C/E45C/W184A/M185A_ hexamer (PDB: 6VKV). SB also binds at the pocket formed by two adjacent CA protomer CA1 (pale yellow) and CA2 (cyan). Relative positions of each CA subunits are indicated as CA1-NTD, CA2-NTD, and CA2-CTD, respectively. **(D)** Cartoon representation of the structure indicating SB’s interactions with the two subunits that form the binding pocket, CA1 and CA2. Hydrogen bonds are denoted by black dashed lines.

To better understand how SA and SB interact with the CA hexamer, we employed induced-fit docking using Swiss-Dock. Figure 5 shows the docked poses of SA or SB within the crystal structure of the CA_A14C/E45C/W184A/M185A_ hexamer. Our results showed that SA (Figure 5A) and SB (Figure 5C) were bound to the pocket formed by two neighboring CA subunits. SA established a hydrogen-bonding network with residues N57, V59, Q63, K70, and N74 of CA1-NTD and Q179 of CA2-CTD (Figure 5B). SB forms hydrogen bonds with the N53, N70, and N74 residues of CA1-NTD, along with the A177 and Q179 residues of CA2-CTD (Figure 5D).

**Figure 6:**
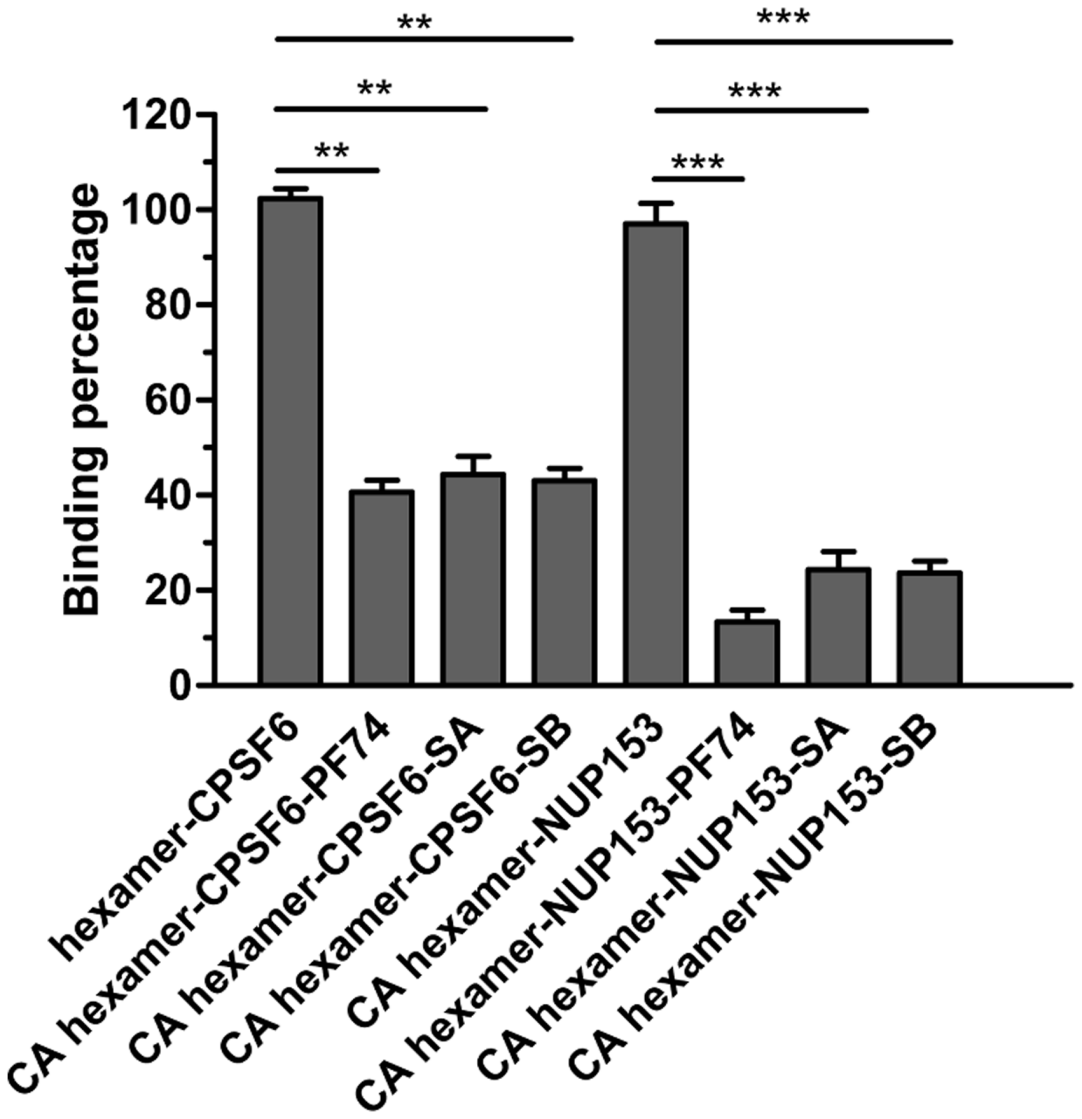
Effect of SA, SB and PF74 on the interaction between hexameric CA and CPSF6_308-327_ or NUP153_1407–1423_. PF74 was used at a concentration of 2.5 μM (approximately 20-fold K_D_), SA at 225 μM (approximately 10-fold K_D_), and SB at 175 μM (approximately 10-fold K_D_) and a final concentration of 5 μM CA hexamer. Biotinylated hexameric CA was firstly loaded to streptavidin-coated biosensors. Then the biosensors were dipped into the plate wells containing CPSF6_308-327_ or NUP153_1407–1423_ in the absence or presence of PF74 or SA or SB. BLI signals were captured by Octet96 system and data were normalized according to the signal in the absence of compound. Comparisons between data were carried out using the paired t-test by GraphPad Prism 5.0. The double asterisk (^**^) and three asterisks (^***^) represent *p* < 0.01 and < 0.001 from the paired *t* test, respectively.

As mentioned previously, this pocket is the binding site for two host cell proteins, CPSF6 and NUP153 (Price et al., 2014). As such, if SA/SB interacts within this pocket it should inhibit the interaction of peptides derived from CPSF6 and NUP153 with hexameric *CA.* Therefore, we synthesized two peptides CPSF6_313–327_ (PVLFPGQPFGQPPLG) and NUP153_1407–1423_ (TNNSPSGVFTFGANSST), both biotinylated at the N-terminus to allow capture on a streptavidin coated sensor chip. Both CPSF6 and NUP153 peptides have been previously demonstrated to interact with hexameric *CA.* We then performed a BLI-based competition assay. CA hexamer at a concentration of 5 μM, either alone or in combination with high concentrations of SA or SB or PF-74, were used to bind to the SA biosensor loaded with biotinylated CPSF6_313–327_ or NUP153_1407–1423._ As Figure 6 showed, PF-74 inhibited, to a degree, the CA hexamer/CPSF6 peptide and the CA hexamer/NUP153 peptide interaction. Our newly discovered compound SA or SB, also inhibited the CA hexamer/CPSF6 peptide and the CA hexamer/NUP153 peptide interaction indicating that it is binding in the pocket formed by two neighboring CA subunits.

### Anti-HIV-1 activity *in vitro*

To further evaluate the antiviral effects of SA and SB, we tested them for protecting C8166 cells from HIV-1-induced CPE. The cytotoxic effect of these compounds on C8166 cells was also assessed. We observed that, SA and SB showed weak inhibitory activity against HIV-1 induced CPE with IC_50_ values of 2.27 μM and 4.73 μM respectively, while the CC_50_ of SA and SB are 22.63 μM and 33.41 μM. The selectivity index of both compounds with antiviral activity was less than 10.

## Discussion

The conformation of HIV-1 CA is critical for the formation of infectious virions (Gross et al., 2000). The binding of a peptide, known as CAI, to a reactive groove located in the CA induces a change in the CA CTD to an assembly-incompetent conformation (Sticht et al., 2005; Ternois et al., 2005; Bartonova et al., 2008), thus making the CAI-binding pocket a potential target for antiviral agents. We previously described a validated approach for the discovery of CAI-binding pocket-targeted inhibitors (Zhang et al., 2019). In the current study, this established method was applied to screen for chemicals that may interact with the CAI-binding pocket in the CTD. We identified two natural products, SA and SB, as previously unknown enhancers of *in vitro* CA assembly.

Previously, it was observed that substitutions of E187A, N183A, Y169A, L211A, or L211S decreased the binding affinity between HIV-1 and peptide CAI (Bartonova et al., 2008). In another study, isothermal titration calorimetry assays revealed that either the N183A or L211S mutation also reduced the binding interactions between HIV-1 CA and compound 16, which binds to the CAI-binding pocket (Machara et al., 2016). As a result, CA with E187A and N183A is assembly competent as the wild-type, and CA with Y169A and L211S is assembly incompetent. BLI analysis conducted in this study revealed that the CA variants mentioned above, except for E187A, exhibited a lower affinity for SA, and all of them showed a lower affinity for SB. Nevertheless, the decrease in binding affinity between the CA variants and compounds in this study was lower than that reported in previous research. As these five mutant residues are located in the CA CTD, it is speculated that SA and SB bind to CA beyond the CA CTD. However, to provide a detailed description of the binding site involved, structural analysis of the complex formed by SA or SB with HIV-1 CA is necessary. This represents a limitation of our study and highlights the need for further research.

**Figure 7:**
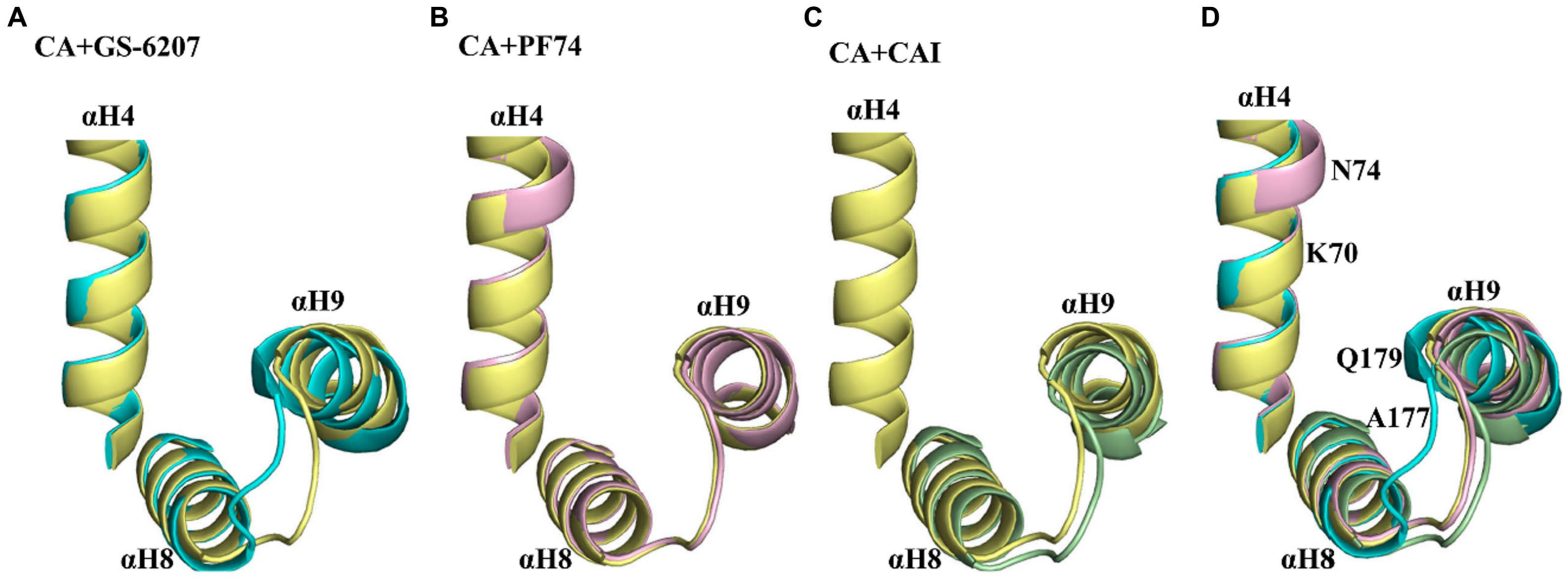
Conformational variability in αH9 of CA hexamers in the absence and presence of different ligands. **(A)** Comparison of αH4, αH8, and αH9 of GS-6207 bound (cyan, PDB:6VKV) vs. unliganded (pale yellow, PDB: 4XFX) CA hexamers. αH9 is positioned closer to αH4 in the presence of GS-6207 compared with native *CA.* **(B)** Comparison of αH4, αH8, and αH9 of GS-6207 bound (light pink, PDB:4XFZ) vs. unliganded (pale yellow, PDB: 4XFX) CA hexamers. αH9 is not positioned closer to αH4 in the presence of PF74 compared with native CA, while several residues in αH4 is positioned closer to αH9 in the presence of PF74 compared with native *CA.* **(C)** Comparison of αH4, αH8, and αH9 of CAI bound (light orange, PDB: 2BUO) vs. unliganded native (pale yellow, PDB: 4XFX) CA hexamers. The αH4 residues in liganded CA hexamer is lacked and are not shown. αH9 is positioned closer to αH4 in native CA compared with in the presence of CAI peptide. **(D)** Superposition of the αH4, αH8, and αH9 from **A–C**. The key residues of CA hexamer interaction with SA or SB are indicated in αH4 and αH9. All comparison were performed with the induced docking PDBs.

Over the past 15 years, a number of compounds have been discovered that target the HIV-1 capsid, as reviewed in reference (McFadden et al., 2021). These compounds either inhibit or increase the assembly of tubular capsid-like particles composed of *CA.* The differing effects on CA assembly may be attributed to conformational changes induced by binding of the compounds (Price et al., 2014). Both PF74 and GS-6207 bind to CA by interacting with two adjacent CA subunits within the hexameric CA, as shown by the structural data. These compounds induce conformational changes in the relative position of αH9 of the CA2-CTD with respect to αH4 of the CA1-NTD. In the presence of GS-6207, αH9 was positioned closer to αH4, while in the presence of PF74, αH4 was positioned closer to αH9 compared to the native CA (Figures 7A,B). Both compounds act as stabilizers to prevent the dissociation of CA subunits from hexamers adjacent to the defect, which is critical for capsid assembly (Bester et al., 2020). Additionally, biophysical analysis suggests that PF74 significantly enhances the resistance of the capsid lattice to mechanical stress and reduces the likelihood of dislocation and material fatigue, thereby enthalpically strengthening the lattice against breakage and disintegration (Domínguez-Zotes et al., 2022). In the case of CAI, its binding to CA results in a conformational change in the CA CTD to accommodate the peptide. This movement leads to the movement of αH9 away from αH4 in CA hexamers (Figures 7C,D), ultimately inhibiting the assembly of capsid-like particles (Ternois et al., 2005). Biophysical analysis also indicated that the binding of CAI-55, which targets the CAI-binding site, increased the intrinsic elasticity and dynamics of the hexagonal lattice. This effect may entropically reduce the probability of CA assembly into a functionally competent conformation (Domínguez-Zotes et al., 2022). In this study, our findings revealed that both SA and SB bound to the CA hexamer. The molecular docking results further indicated that they bound to the cavity created by two neighboring protomers within the CA hexamer by interacting with key residues located in αH9 and αH4 of *CA.* These observations provide insights into the mechanisms underlying the effects of SA and SB on CA assembly, such as promoting multimerization. Notably, our findings are consistent with those of previous studies.

SA is a natural compound derived from *Rheum officinale* Baill and *Rheum palmatum* L. and is known for its laxative properties and potential pharmacological effects such as anti-obesity, hypoglycemic, hepatoprotective, anti-inflammatory, and anti-cancer activities (Le et al., 2021). In addition to these, SA also inhibits various viral enzymes including HIV-1 reverse transcriptase-associated DNA Polymerase (IC_50_ = 5.3 μM), RNase H (IC_50_ = 1.9 μM), integrase (IC_50_ = 3.8 μM), and protease of zika virus (IC_50_ = 0.66 μM) (Esposito et al., 2016; Coelho et al., 2022). In cell-based assays, SA demonstrated anti-HIV-1 activity, with an EC_50_ of approximately 9 μM (Esposito et al., 2016). SB, which has a similar anthraquinone structure to SA, has been identified as an inhibitor of the SARS-CoV-2 main protease with an IC_50_ of 104 nM (Abdallah et al., 2021). In this study, SA and SB were identified as weak inhibitors of HIV-1 replication and enhancers of HIV-1 CA multimerization via binding to the CA NTD/CA CTD interface formed by two neighboring monomeric CA within the CA hexamer. Both compounds contain two anthraquinone cores with two glycoside groups, which distinguishes them from previously described CA modulators (Xu et al., 2016; Sun et al., 2020). It is not yet clear whether SA or SB are capsid inhibitors *in vivo*, thus highlighting the need for further study. Nevertheless, these compounds show promise as leads for the design of drugs targeting HIV-1 CA assembly.

## Conclusion

Our study revealed that SA and SB were potent modulators of HIV-1 CA assembly. These compounds possess an anthraquinone core and exhibit unique structures that differ from those of the previously reported CA modulators. Despite not being new compounds, SA and SB have demonstrated promising results in modulating HIV-1 CA assembly and may serve as starting points for the design of novel drugs. To further explore anthraquinone core as a potential scaffold for CA assembly modulation, other compounds with similar structures should be tested. Additionally, determining the complex structure of SA or SB with the HIV-1 CA hexamer will provide insights into their binding sites in *CA.* Overall, our study highlights the potential of natural products, such as SA and SB, to discover novel anti-HIV-1 inhibitors with unique structures.

## Data availability statement

The original contributions presented in the study are included in the article/supplementary material, further inquiries can be directed to the corresponding author.

## Author contributions

D-WZ: Conceptualization, Methodology, Software, Writing – original draft, Writing – review & editing. X-SX: Data curation, Investigation, Visualization, Writing – review & editing. RZ: Writing – review & editing. ZF: Supervision, Writing – review & editing.

## Funding

The author(s) declare financial support was received for the research, authorship, and/or publication of this article. This study was supported by the National Natural Science Foundation of China (No. 31700297) and the National Compound Resource Center (Shanghai, China) for supplying the small compounds used in this study.

## Conflict of interest

The authors declare that the research was conducted in the absence of any commercial or financial relationships that could be construed as a potential conflict of interest.

## Publisher’s note

All claims expressed in this article are solely those of the authors and do not necessarily represent those of their affiliated organizations, or those of the publisher, the editors and the reviewers. Any product that may be evaluated in this article, or claim that may be made by its manufacturer, is not guaranteed or endorsed by the publisher.

## Footnotes

^1^ http://www.pymol.org
